# Inaugural Address Delivered by Prof. James P. White, at the Opening Session of the State Medical Society, in Albany, Feb. 1, 1870

**Published:** 1870-02

**Authors:** 


BUFFALO

### Medical and Surgical Journal.

VOL. IX. FEBRUARY, 1870. No. 7.

### Original Communications.

ART. I.—*Inaugural Address delivered by* PROF. JAMES P. WHITE, *at the opening Session of the State Medical Society, in Albany, Feb.* 1, 1870.

(Reported for the Buffalo Medical and Surgical Journal.)

GENTLEMEN :—

Permit me to congratulate you upon being again assembled, and with increased numbers, for renewed social intercourse, and for the discussion of scientific subjects which concern the medical profession. In taking the chair, which has been occupied by so many distinguished predecessors, I should do injustice to my own feelings, did I not, in the first place, thank you most sincerely, for having by your suffrages elevated me to this high position. Whilst assuring you that, whatever a grateful heart, and an ardent zeal can accomplish in promoting the objects for which this venerable society is assembled, it is but truth to admit that the uninterrupted pursuit of professional duties, has not furnished the parliamentary training necessary for the prompt discharge of the duties devolving upon your presiding officer. I must therefore bespeak your considerate forbearance, and express the hope, that by your counsel and indulgence we shall be able to bring our labors to a satisfactory conclusion, though strict rules of order may sometimes be unconsciously violated.

According to a by-law of this society, the president is required at the opening of the session, briefly to present the condition of the medical profession in the State; and to make such suggestions as may seem proper or necessary touching the interests of medicine, and its relations to the public.

The Medical Schools of the State, may now be said to be in a highly satisfactory condition. They are yearly increasing their requirements for graduation—though the standard is undoubtedly still too low—demanding constant effort on the part of all men of progress, to carry it still higher.

The different institutions are yearly enlarging their facilities for instruction, and in a laudable competition for increased classes, are affording opportunities in both didactic, and clinical teaching, which the student will scarcely find surpassed in any part of Europe.

There is however one step in a forward direction, which should now be taken, and which it seems to me this society can promote by its influence, and endorsement.

I allude to the teaching of Psychological Medicine, as a part of the curriculum of the college course. Is it less important that the diseases of the mind, always involving the brain and nervous system, with their sympathetic connexions, and influences, should be properly taught and illustrated with cases, than any of those maladies, now deemed essential to be clinically taught, in every institution? The attention of the profession is beginning to receive this direction—some steps have already been taken toward its accomplishment, and I believe the medical mind is thoroughly ripe for action, if commended by this society in suitable and proper terms.

Permit me therefore to suggest the appointment of a select committee, to take this matter duly into consideration, and recommend to the society, what action, if any, it should take in its furtherance.

To the Medical Journals have been added, during the last year, "Archives of Opthalmology and Otology," and "The American Journal of Syphilography, and Dermatology."

Those publications, to which attention was very properly called by my predecessor, continue without change, to furnish, in a highly creditable manner, all the novelties of interest, and I believe it may be added, that judging from the increased support which they receive, the profession is becoming more and more appreciative of their usefulness and value to the practitioner.

At the last meeting of the American Medical Association at New Orleans, in May last, a committee was appointed, with instructions to reprint, through the Committee of Publication—the Latin and English portions of the "Provisional Nomenclature of the Royal College of Physicians of London," and to distribute it under the designation of the "Proposed Nomenclature." This Committee was desired to report at the next meeting, what alterations, if any, are necessary to adapt the proposed nomenclature to general use in the United States.

It is hoped, and expected, that such a report will be reached as will be adopted by the entire medical profession of the United States, as well as by the medical departments of the Army and Navy, general hospitals, boards of health, and the census bureau. The importance of such a uniform system can scarcely be overestimated, and need not be dwelt upon here.

To the end that such nomenclature if adopted in May next, by the representatives of the profession from all the States, may be made available in taking the next census in this State, thereby greatly increasing the value of its statistics, I would suggest that a Committee be appointed by this Society, with power, if such nomenclature be approved by them, to publish it immediately and distribute it throughout the State, through the agency of the County Societies.

In this way, we shall anticipate, by more than a year and a half, the time when it would be received if delayed for the published transactions of the next annual meeting of this Society. In this way only can it be made useful in making the census reports of 1870.

Your Committee might also be instructed to act for this Society, in compliance with the request of the Committee in behalf of the American Medical Association, in making such suggestions as would promote perfection in the arrangement and details of the nomenclature.

It will be proper also for this Society, upon the recommendation of the Committe on Pharmacology, to appoint three delegates to represent its interests at the meeting of the National Medical Convention, for the revision of the United States Pharmacopœia, to be held in Washington, on the first Wednesday of May next.

I would also suggest that a more catholic influence might be exerted upon the profession of this country, at the same time giving opportunity for free interchange of views and opinions, by increasing the number of delegates sent by this Society, to the meetings of kindred societies in neighboring States. In this connection, I may mention, that with several other members of this Society, I accepted an invitation to attend the semi-annual meeting of the Medical Society of the State of Pennsylvania, held in Erie during the month of June last. I should do injustice to my own feelings did I not gratefully acknowledge the kindness and hospitality extended to us by the vigorous and useful sister society of that great State.

There is a crying evil which I wish it were practicable for this Society to lessen—most severely felt in all the small towns, and rural districts in the State, by junior members of the profession.

Allusion is here made to the low rate of compensation for medical services. In addition to a large modicum of gratuitous labor, every where given to the poor, and the clergy, the physician finds himself working very hard, night and day, with scarcely sufficient revenue to respectably support and educate his family. Obliged to economize in every direction, the ambitious young man is compelled to forego the purchase of such books, and instruments, as are requisite to his professional advancement. It often occurs in a very extensive consultation practice, in the counties in the Western portion of the State, that I find the livery-man charging as much for an ordinary conveyance to the house of the patient, as the medical attendant receives for his services, traveling over the same route in his own conveyance.

This lamentable condition has arisen frequently from the fact, that the old practitioner or pioneer in the profession, having become forehanded, still lives to compete in the race, and deriving his support chiefly from his farm, continues to charge the prices which he established half a century since.

Whether this Society can do anything to remedy or mitigate the embarrassment of many a high-spirited but illy requited laborer, remains for your deliberate consideration. Could all the county societies be induced to adopt and adhere to a reasonable tariff of charges, the correction of this grievance could be easily accomplished. This schedule of prices, sanctioned by the fellow-practitioners of the county, could be referred to by the physician, instead of the habitual charges of his antiquated neighbor, in settling all questions of finance with his patients. The expense of living, the cost of library and apparatus, have greatly increased within a few years, and the fees for services have not advanced, certainly not in the same ratio. Many an aspiring practitioner who would be a useful member of this Society, and be profited by listening to its proceedings, is debarred the privilege of attendance by the inadequacy of his income. Can this Society point out a remedy for this unfortunate state of things with our brethren?

The present age is one remarkable, not only for the advancement of all departments of human knowledge, but for private liberality in promoting scientific investigations.

The generous presentation last year, of one hundred dollars to the Prize Committee of this Society, by Dr. Hiram Carliss, for the best essay on Tubercular Consumption, is evidence that this spirit of benevolence actuates our own noble profession, and that that enthusiasm which is the strength of all true science is not dimmed by age or years of professional toil. Others I trust may be led to imitate the examples of the Fathers in bestowing means for the promotion of medical science—stimulating the labors of the diligent student by the hope of reward, as well as the honor of success in his investigations.

During the last year death has deprived us of some of our most active and able members. On the 30th day of March last, Alexander H. Stevens departed this life at the ripe age of more than seventy-nine years. On the 18th day of April, Alden March followed his distinguished fellow-surgeon, at the age of seventy-four years. These great men, who had few equals and no superiors will be mourned by the whole profession. These distinguished men had, both been Presidents of the American Medical Association, had both occupied the highest positions in the gift of this Society, and had both been, for a long period, favorite teachers of operative surgery. But, gentlemen, assured that suitable action will be taken to record their illustrious deeds and commemorate their worth, and conscious of my inability to do justice to the subject, I will not attempt their eulogy.

Prof. James Hadley, an honorary member of this Society—for more than half a century a teacher of Chemistry—a man without guile, from whose able lectures I received my first lessons in this department, died in July last at the advanced age of eighty-four years, leaving a large circle of pupils and friends to mourn his loss. Alexander Thompson, an ex-president of this Society, who was always punctual in his attendance upon its meetings and actively participated in its proceedings, and who leaves a vacancy which it will be difficult to supply, died in June last. The Society has also lost from its ranks since our last meeting, Minturn Post, who died April 26th, 1869, and Dr. Thomas Cook, the associate of Mott and Stevens, and perhaps others whose deaths have not been brought to my attention, all should receive suitable commemorative notice.

Although dissection is now legalized in this and most of the other states in the union, and its necessity and propriety no longer questioned, it is, nevertheless, a lamentable fact that provision for the supply of subjects is but inadequately made, often rendering it necessary to resort to improper means to make up the deficiency. By a law ot the State, a convict dying in one of the State Prisons, whose body is not claimed by his friends in twenty-four hours, is to be given to the Medical Colleges for dissection. There are at this time several Penitentiaries in the state to which the same law might with equal propriety be made applicable. At the penitentiary in Buffalo which comes more especially under my own observation, there have been, as I am informed by Dr. Ring, the attending physician, about a dozen deaths during the past year. Nearly all of them persons without friends or relatives—prostitutes and criminals of various grades. Their burial, in nearly every case, is at the public charge. It would seem that science should have the benefit of the bodies for scientific purposes of those who have been supported during life by the public bounty and who have no kindred surviving to claim them. Would it not, therefore, be proper for this society, to petition the Legislature to make the present law in regard to those dying in State Prisons applicable and mandatory on the Superintendents of the Penitentiaries?

Again, bespeaking your indulgence for any short comings on the part of your presiding officer, without detaining you longer with suggestions, I beg to announce that the society is now organized and ready for business.

